# Restoration of PPD-Induced Injury in iHDPCs via the OS–Mitochondrial Apoptosis Pathway: Screening and Validation of Natural Compounds

**DOI:** 10.3390/ijms27209109

**Published:** 2026-10-14

**Authors:** Yi Ren, Jiqing He, Linxin Zheng, Lizhi Yue, Congfen He, Dianchao Huang, Fenglan Zhang

**Affiliations:** 1 School of Light Industry Science and Engineering (International School of Cosmetics), Beijing Technology and Business University, Beijing 100048, Chinayuelizhi11@163.com (L.Y.); hecf@th.btbu.edu.cn (C.H.); 2 National Institutes for Drug Control, Beijing 100050, China

**Keywords:** PPD, human immortalized hair follicle cells, OS–mitochondrial apoptosis, hair dye

## Abstract

This study aimed to investigate the protective effects of curcumin (Cur), resveratrol (Res), and astragaloside IV (AS-IV) against p-phenylenediamine (PPD)-induced injury in human immortalized dermal papilla cells (iHDPCs), with a focus on phenotypic endpoints that are suggestive of the oxidative stress–mitochondrial apoptosis pathway. Putative core targets were predicted via network pharmacology and molecular docking, and cellular responses were evaluated using CCK-8, DCFH-DA, JC-1, and Annexin V/PI assays in both PPD- and H_2_O_2_-exposed models. 1623 Collectively, these observations are consistent with the possibility that Cur, Res, and AS-IV may alleviate PPD-induced cellular damage, potentially through modulation of oxidative status and mitochondrial homeostasis. However, because the current experiments do not directly measure NRF2 nuclear translocation, downstream antioxidant enzyme expression (e.g., P-AKT HO-1, NQO1), BAX/BCL-2 ratio, cytochrome c release, or caspase activation, they cannot establish target engagement or a definitive causal link to the proposed pathway. Therefore, our findings should be interpreted as correlative and hypothesis-generating; direct mechanistic validation is required to confirm the involvement of this pathway. Nevertheless, the consistent protective phenotypes observed make these compounds, particularly AS-IV, promising candidates for the development of next-generation hair dyes.

## 1. Introduction

Over the past two decades, with the shift in aesthetic concepts, the practice of hair dyeing has significantly increased, leading to more and more regulatory scrutiny of the safety of oxidative hair dyes. Among the various chemical intermediates commonly used in permanent hair dye formulations, p-phenylenediamine (PPD) has become the most widely used primary intermediate due to its ability to form stable color groups during the oxidation process [1]. However, this chemical activity that ensures a good coloring effect also leads to the clear documentation of its toxicological characteristics. PPD is a potent sensitizing agent, which can lead to a large number of allergic contact dermatitis cases [2], and it also has strong cytotoxicity towards hair follicle cells, especially the hair follicle papilla [3]. The hair follicle papilla is a special mesenchymal microenvironment that regulates hair morphology, periodic growth and regeneration [4]. Therefore, maintaining the functional integrity of this structure is crucial for healthy hair growth, and any chemical damage to this structure may cause impaired hair follicle function.

Currently, an increasing number of experiments have shown that the main mechanism by which PPD causes damage to dermal papilla cells is the production of excessive reactive oxygen species (ROS) [3]. Excessive ROS activates the initiation of OS and triggers a series of molecular reactions, ultimately leading to mitochondrial damage. Excessive accumulation of ROS within mitochondria causes a decrease in mitochondrial membrane potential (ΔΨm), a change in permeability transition pore opening, and the release of pro-apoptotic factors such as cytochrome c [5]. Additionally, the balance between pro-survival Bcl-2 family members and pro-apoptotic effect proteins (such as BAX) also affects the apoptotic pathway [6]. Once this balance shifts towards the oligomerization of BAX, the apoptotic process becomes irreversible. Therefore, the OS–mitochondrial apoptosis axis constitutes a key pathogenic cascade from PPD exposure to hair follicle cell detachment [7]. Although the subtoxic PPD concentration (IC_20_) in vitro is much lower than the 2% regulatory limit for hair dyes, direct comparison is limited by differences in exposure conditions. In vivo, dermal papilla cell exposure is modulated by formulation chemistry, scalp barrier properties, and follicular delivery, which are not captured in cell culture. These data suggest that repeated subtoxic PPD exposure may cause cumulative oxidative damage, but further studies with physiologically relevant models are needed to assess its toxicological significance.

The compounds found in natural plants have attracted widespread attention due to their structural diversity, strong multi-target regulatory capabilities, and the proven good tolerance in previous literature. Cur, a polyphenolic substance extracted from turmeric, has been extensively studied for its excellent antioxidant and anti-inflammatory properties. The latest evidence indicates that it can upregulate NRF2-driven detoxification enzymes and stabilize mitochondrial function [8,9]. Res, an anti-toxin found in grapes and berries, is also renowned for its ability to activate Sirtuin proteins and maintain mitochondrial homeostasis under OS conditions [10]. Res and its derivative, quassin, have also been proven to improve mitochondrial redox homeostasis and function through the SIRT1 signaling pathway, thereby alleviating intestinal damage caused by OS [11,12]. AS-IV, a triterpenoid saponin extracted from the traditional Chinese medicine Astragalus [13], exhibits significant cellular protective effects in the cardiovascular and nervous systems, mainly through activating the NRF2–HO-1 antioxidant axis and inhibiting BAX-mediated mitochondrial apoptosis [14]. N-acetylcysteine (NAC), a synthetic thiol precursor that can be converted into reduced glutathione, has long been proven to be a direct free radical scavenger and can indirectly enhance the endogenous glutathione pool [15]; its ability to replenish intracellular sulfhydryl reserves and alleviate oxidative damage makes it a standard drug in experimental and clinical studies of chemical-induced cytotoxicity, although its mechanism of action is mainly a stoichiometric effect rather than transcriptional regulation [16].

Despite the above literature studies, there are currently no studies specifically comparing the repair effects of these three compounds on the damage caused by PPD to immortalized human dermal papilla cells. Moreover, the relative binding interactions between them and the core regulatory nodes of the OS–mitochondria pathway, particularly NRF2, BAX, and Bcl-2, have not been systematically studied. This lack of direct comparison makes it impossible for us to determine which compound has a more superior protective effect and which molecules play a role in repairing the cells.

Given this gap, this study aimed to evaluate and compare Cur, Res and AS-IV in repairing OS induced by PPD in human immortalized dermal papilla cells (iHDPCs), with a particular focus on the OS–mitochondrial apoptosis pathway [17]. Our research methods combine target prediction in network pharmacology, molecular docking simulations, and a series of comprehensive cellular function assays, including cell viability, intracellular reactive oxygen species (ROS) quantification, mitochondrial membrane potential assessment, and apoptosis feature analysis [18]. We aimed to explore which specific molecules these compounds use to repair the cells, ultimately converging on the NRF2–BAX–Bcl-2 regulatory axis, and investigate whether the differences in binding affinity lead to different protective effects. Through a systematic comparison framework, this study not only helps to clarify the mechanism basis of the protective effects of these natural substances, but also provides preclinical evidence for the next generation of hair dye components, achieving a synergistic balance between effective coloring and protection of the hair follicles.

## 2. Results

### 2.1. Network Pharmacology Analysis Predicts Shared Targets in the OS–Mitochondrial Apoptosis Pathway

To systematically identify the candidate targets through which OS modulates mitochondrial apoptosis and hair follicle pathology, a standardized network pharmacology pipeline was implemented. Bioactive constituents of OS were retrieved from TCMSP (https://old.tcmsp-e.com/tcmsp.php, accessed on 1 January 2026), BATMAN-TCM (http://bionet.ncpsb.org.cn/batman-tcm/) (accessed on 1 January 2026), and SwissTargetPrediction (http://www.swisstargetprediction.ch/), with compounds filtered by oral bioavailability (OB ≥ 30%) and drug-likeness (DL ≥ 0.18) criteria where available; SwissADME (http://www.swissadme.ch/) was consulted for constituents lacking TCMSP annotations. Putative protein targets of these qualified compounds were predicted via SwissTargetPrediction (with Homo sapiensas the species setting) and cross-referenced with SEA (https://sea.bkslab.org/), then standardized to official gene symbols and UniProt accession IDs (https://www.uniprot.org/) by removing duplicates, non-human entries, and ambiguous records, yielding a total of 221 component-derived targets.

Disease-related targets were collected from GeneCards (https://www.genecards.org/), OMIM (https://omim.org/), DisGeNET (https://www.disgenet.org/), TTD(http://db.idrblab.org/ttd/) (accessed on 1 January 2026), and PharmGKB (https://www.pharmgkb.org/) by searching a consolidated keyword set that centered on “hair follicle damage” and “oxidative stress–mitochondrial apoptosis” as the two core pathological axes, supplemented with related terms including “alopecia,” “androgenetic alopecia,” “ROS,” “mitochondrial dysfunction,” “cytochrome c release,” “BAX/BCL-2” and “hair cycle regression”; targets with a GeneCards relevance score ≥ median or a DisGeNET confidence score ≥ 0.10 were retained, duplicates were removed, and all entries were unified to Homo sapiensgene symbols via UniProt, resulting in a total of 2135 disease-associated targets.

**Figure 1:**
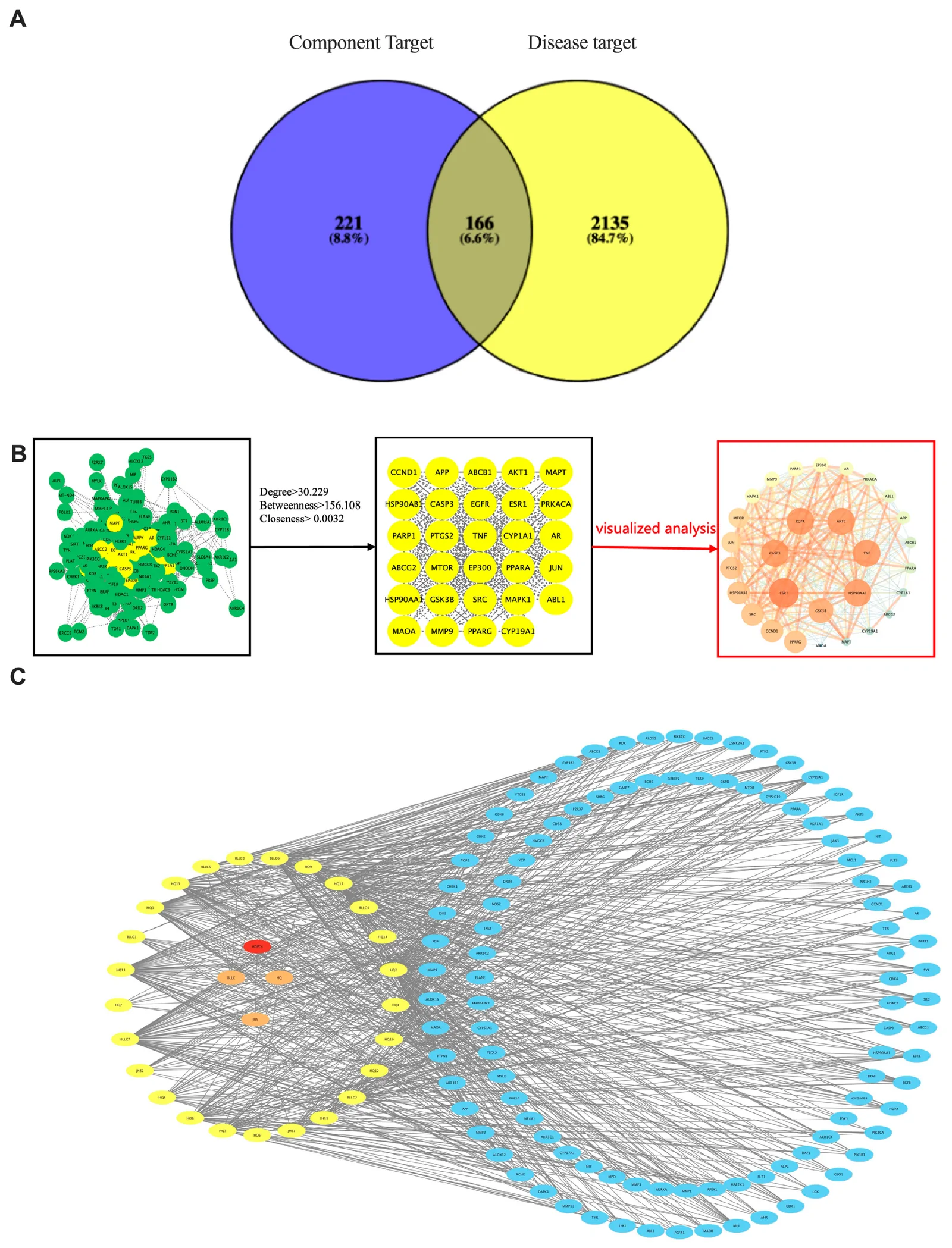

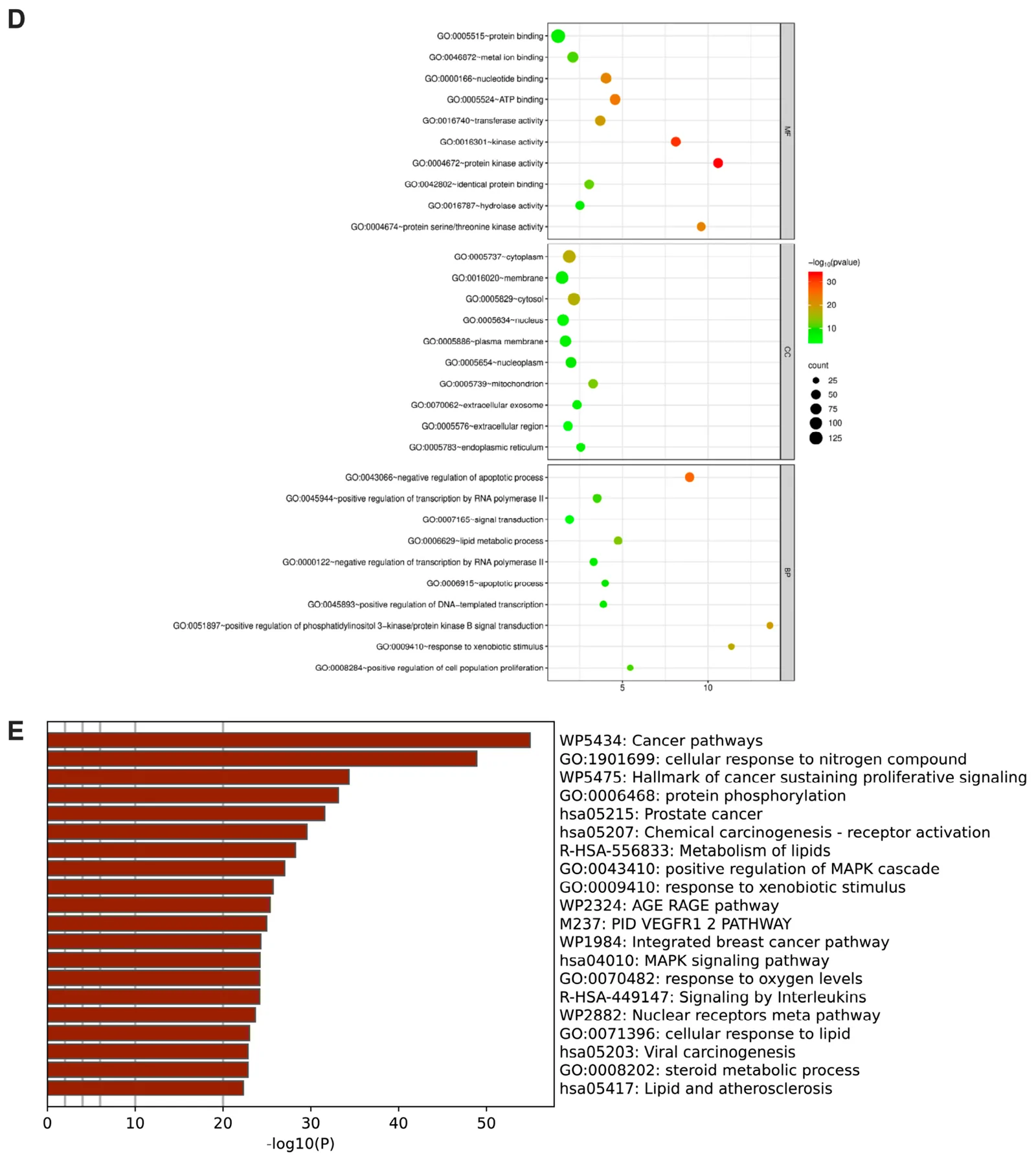
Network pharmacology–based identification of shared targets between natural compounds (Cur, Res, AS-IV) and hair follicle injury with oxidative stress–mitochondrial apoptosis, and their functional enrichment. (**A**) Venn diagram showing 166 overlapping targets between natural compounds (Cur, Res, AS-IV) and hair follicle injury. (**B**) Protein–protein interaction (PPI) network of 166 intersection targets. (**C**) Workflow of core target screening and visualization analysis. (**D**) GO enrichment bubble chart showing top 10 biological processes. (**E**) KEGG pathway enrichment analysis showing top 10 pathways.

The component-derived and disease-associated target lists were intersected using Venny 2.1 (https://bioinfogp.cnb.csic.es/tools/venny/) (accessed on 20 January 2026) and custom R scripts, yielding 166 shared targets (Figure 1A), which were defined as the final core target set for downstream analysis. The PPI network of these shared targets was constructed using STRING (version 11.5; https://string-db.org/) with H. sapiens selected, a minimum interaction score of 0.400, and both experimental and text-mining evidence enabled; isolated nodes were hidden. The network was imported into Cytoscape (version 3.9.1; https://cytoscape.org/) for topological analysis using NetworkAnalyzer, and targets exceeding the thresholds of degree > 30.229, betweenness centrality > 154.166, and closeness centrality > 0.0032 were extracted as hub targets (Figure 1B). A component–target network was additionally visualized in Cytoscape to map OS ingredients to these hubs (Figure 1C).

GO and KEGG enrichment analyses were performed on the 166 shared targets using DAVID (version 6.8; https://david.ncifcrf.gov/) and the R package clusterProfiler (v4.0), with FDR < 0.05 as the significance cutoff. GO terms (Figure 1D) were enriched in biological processes including response to ROS/oxygen/lipid/xenobiotic stimulus, regulation of mitochondrial apoptosis, protein phosphorylation, and MAPK cascade; molecular functions such as protein/ATP/kinase binding and caspase activity; and cellular components including mitochondrion, membrane, nucleoplasm, and cytoplasm. KEGG pathways (Figure 1E) prominently featured PI3K–AKT, MAPK, p53, apoptosis, AGE–RAGE, VEGF, and steroid hormone signaling. Collectively, these results position the OS-intersected targets at the mechanistic interface of hair follicle damage and oxidative stress–driven mitochondrial apoptosis, providing a prioritized candidate pool for subsequent molecular docking and in vitro/in vivo validation.

### 2.2. Molecular Docking Confirms High Binding Affinity to Core Targets

**Figure 2:**
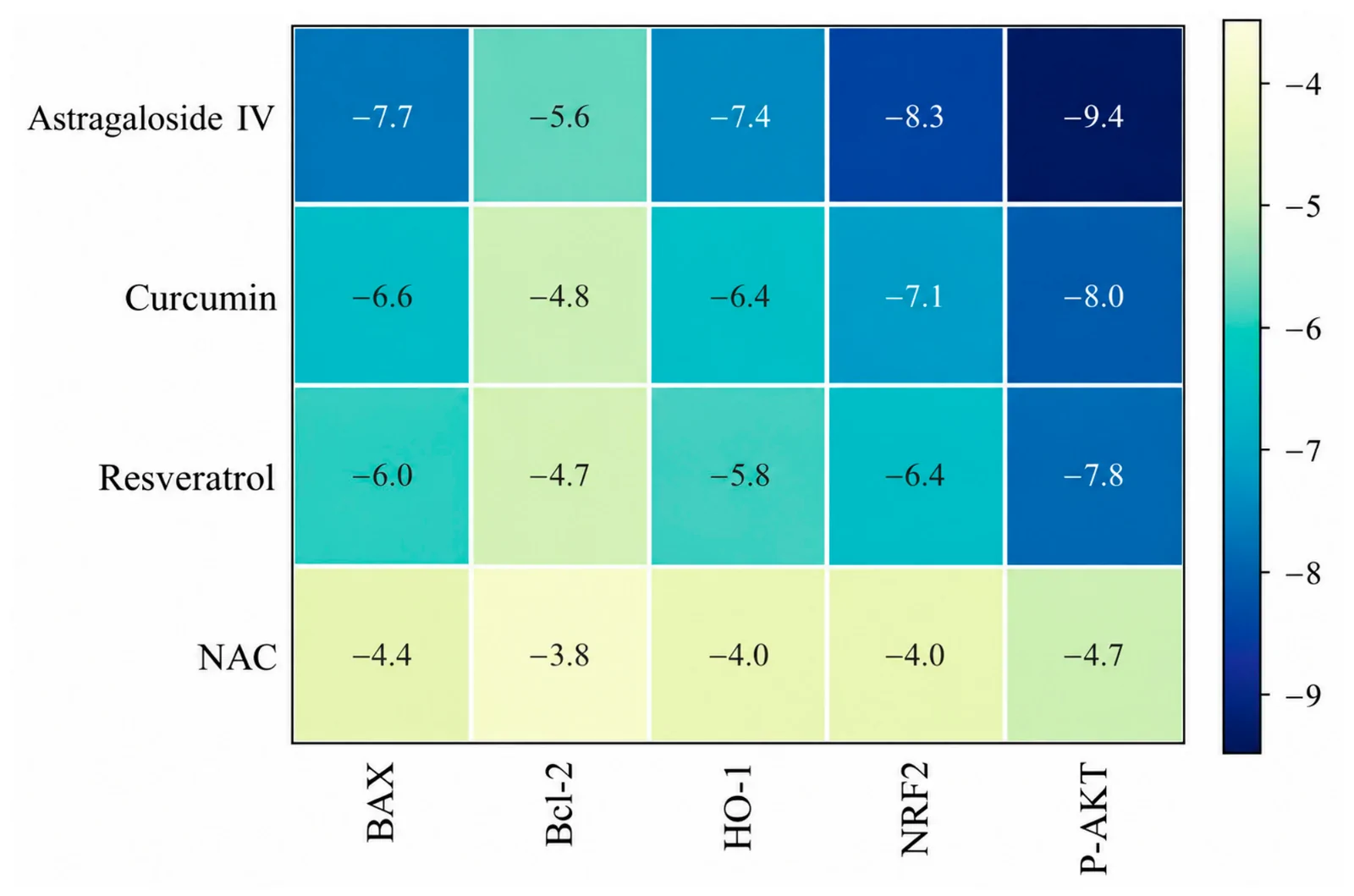
Heatmap of molecular docking binding affinities. Binding energies (kcal/mol) of Cur, Res, AS-IV, and NAC with BAX, Bcl-2, HO-1, NRF2 and P-AKT. Blue represents stronger binding (more negative values); yellow represents weaker binding.

**Figure 3:**
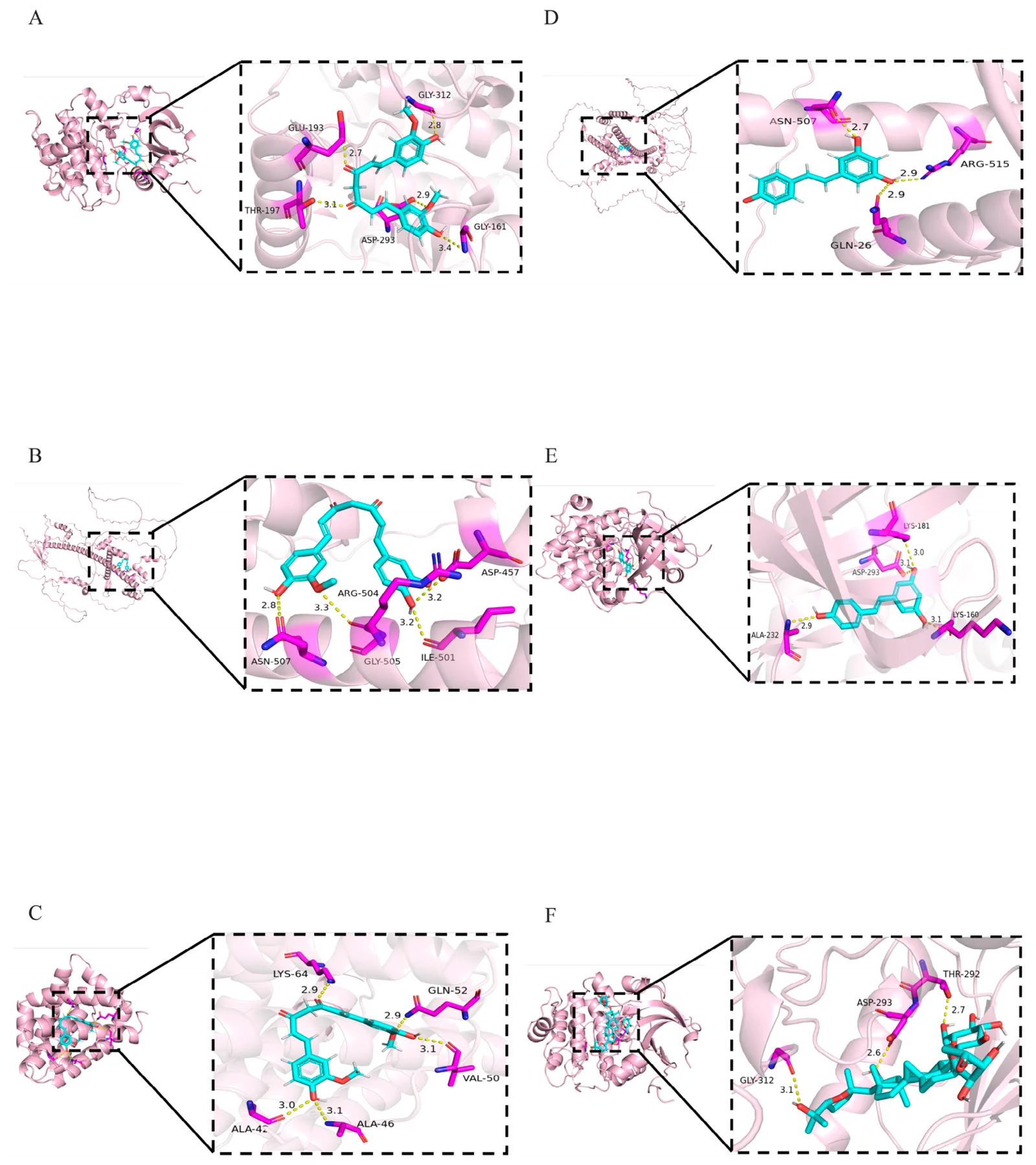
Molecular docking interaction modes of Cur, Res, and AS-IV with core target proteins. (**A**) Cur binding to P-AKT via hydrogen bonds with GLU-193, GLY-312, ASP-293, THR-197, and GLY-161. (**B**) Cur binding to NRF2 via hydrogen bonds with ASN-507, GLY-505, ASP-457, ILE-501, and ARG-504. (**C**) Cur binding to BAX via hydrogen bonds with GLN-52, LYS-64, ALA-42, ALA-46, and VAL-50. (**D**) Res binding to NRF2 via hydrogen bonds with ASN-507, GLN-26, and ARG-515. (**E**) Res binding to P-AKT via hydrophobic interaction with ALA-232 and hydrogen bonds with LYS-181, LYS-160, and ASP-293. (**F**) AS-IV binding to P-AKT via hydrogen bonds with ASP-293, THR-292, and GLY-312. Hydrogen bonds are shown as yellow dashed lines.

Molecular docking was performed to evaluate the binding affinities of Cur, Res, AS-IV, and NAC (positive control) to five core targets: NRF2, BAX, Bcl-2, HO-1, and P-AKT. The binding affinity heatmap (Figure 2) showed that the four compounds exhibited varying binding affinities across the targets, with some binding energies below −7.0 kcal/mol. Notably, Cur, Res, and AS-IV all showed their strongest binding affinity to P-AKT, while NAC showed equal affinity to NRF2 and P-AKT. AS-IV demonstrated the highest overall affinity to P-AKT (−9.4 kcal/mol), outperforming Cur (−8.0 kcal/mol), Res (−7.8 kcal/mol), and NAC (−4.7 kcal/mol). Furthermore, the compounds showed differential binding preferences: AS-IV also showed strong binding to NRF2 (−8.3 kcal/mol), BAX (−7.7 kcal/mol), and HO-1 (−7.4 kcal/mol); Cur showed stronger binding to NRF2 (−7.1 kcal/mol) and HO-1 (−6.4 kcal/mol); Res showed stable binding to NRF2 (−6.4 kcal/mol) and HO-1 (−5.8 kcal/mol) but relatively weaker binding to BAX (−6.0 kcal/mol) and Bcl-2 (−4.7 kcal/mol). The interaction mode diagrams (Figure 3A–F) further revealed the detailed binding mechanisms. Cur formed hydrogen bond networks with key residues of multiple targets, including GLU-193, GLY-312, ASP-293, THR-197, and GLY-161 in P-AKT, ASN-507, GLY-505, ASP-457, ILE-501, and ARG-504 in NRF2, as well as GLN-52, LYS-64, ALA-42, ALA-46, and VAL-50 in BAX. Res exhibited a combination of hydrophobic interactions and hydrogen bonds, including hydrophobic interaction with ALA-232 and hydrogen bonds with LYS-181, LYS-160, and ASP-293 in P-AKT, and hydrogen bonds with ASN-507, GLN-26, and ARG-515 in NRF2. AS-IV showed the highest reliance on dense hydrogen bond networks, forming hydrogen bonds with ASP-293, THR-292, and GLY-312 in P-AKT. These results indicate that Cur, Res, and AS-IV are capable of stably binding to the active pockets of core targets through multi-modal interactions, with AS-IV exhibiting the most extensive hydrogen bonding network and highest overall binding affinities, particularly to P-AKT.

### 2.3. PPD and H_2_O_2_ Induce Concentration-Dependent Cytotoxicity in iHDPCs

**Figure 4:**
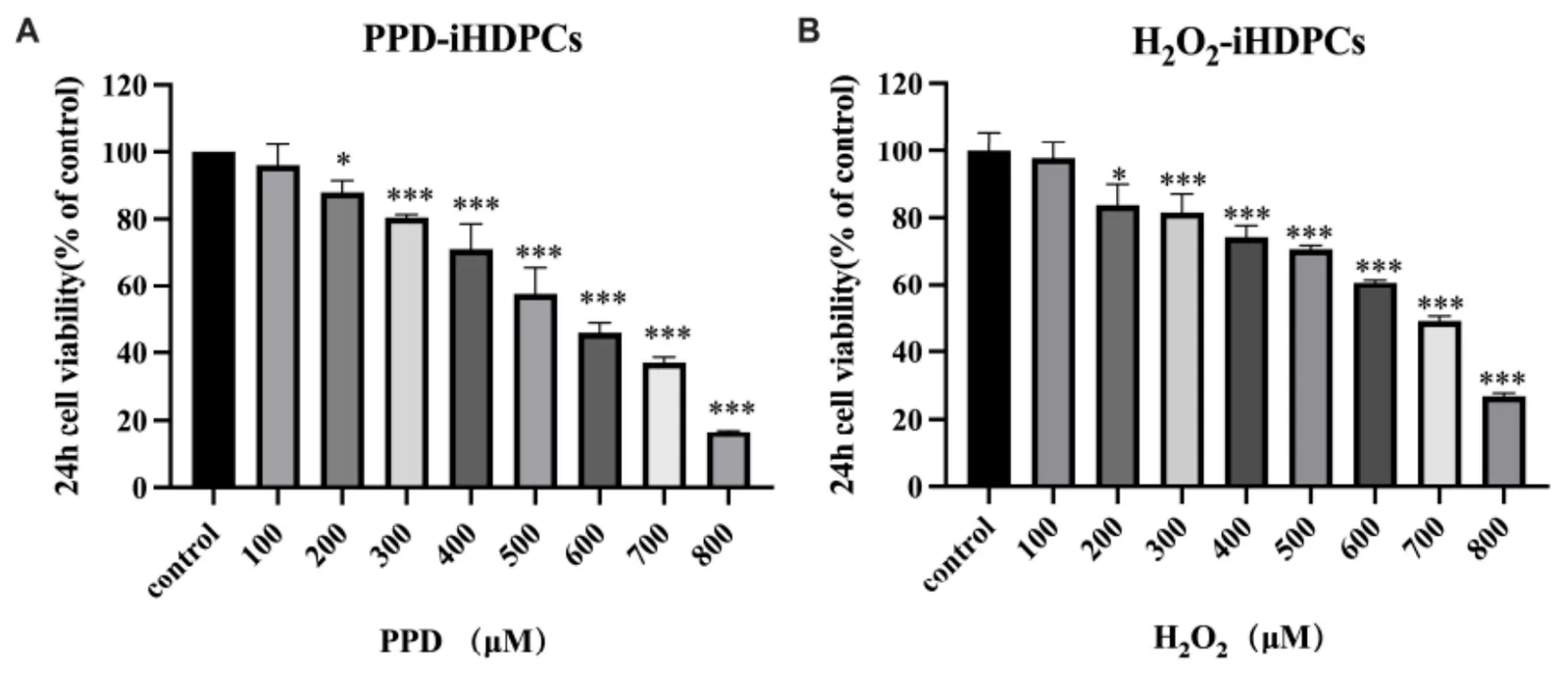
Cytotoxicity of PPD and H_2_O_2_ in iHDPCs. (**A**) CCK-8 assay showing iHDPC viability after 24 h treatment with graded concentrations of PPD (0–800 μM). (**B**) CCK-8 assay showing iHDPC viability after 24 h treatment with graded concentrations of H_2_O_2_ (0–800 μM). Data are presented as mean ± SD *(n* = 3). Data were analyzed by one-way ANOVA followed by Tukey’s post hoc test. * *p* < 0.05, *** *p* < 0.001 vs. control.

The cytotoxic effects of PPD and H_2_O_2_ on iHDPCs were assessed after 24 h treatment. As shown in Figure 4A, PPD reduced cell viability in a dose-dependent manner. The 20% inhibitory concentration (IC_20_) was approximately 300 μM, and the 50% inhibitory concentration (IC_50_) was approximately 600 μM. For context, 300 μM is substantially lower than the 2% (*w*/*v*) maximum concentration permitted for oxidative hair-dye ingredients by the Cosmetic Safety Technical Specification (2015 edition); however, a direct comparison between this in vitro concentration and the regulatory limit is not straightforward, as the actual exposure of dermal papilla cells in vivo depends on formulation chemistry, oxidative activation, dilution, scalp barrier properties, rinsing, and follicular delivery—factors that are not recapitulated in cell culture. At the IC_20_ (300 μM), PPD still induced a measurable suppression of metabolic activity. Similarly, H_2_O_2_ treatment resulted in a concentration-dependent decrease in viability (Figure 4B), with an IC_20_ of approximately 300 μM and an IC_50_ of approximately 600 μM (corresponding to ~0.00204%).H_2_O_2_ exhibited detectable cytotoxicity at 200 μM, showing earlier damage effects compared to PPD, consistent with its role as a classic OS inducer. Based on these results, 300 μM PPD was selected as the sub-toxic insult for subsequent repair experiments, and H_2_O_2_ concentrations of 100, 300, and 600 μM were used to create graded OS conditions. These findings confirm that both PPD and H_2_O_2_ are effective in establishing an oxidative damage model in iHDPCs, with the IC_20_ concentration representing a sublethal insult suitable for evaluating protective interventions.

### 2.4. Cur, Res, and AS-IV Are Non-Toxic Within Defined Concentration Ranges

**Figure 5:**
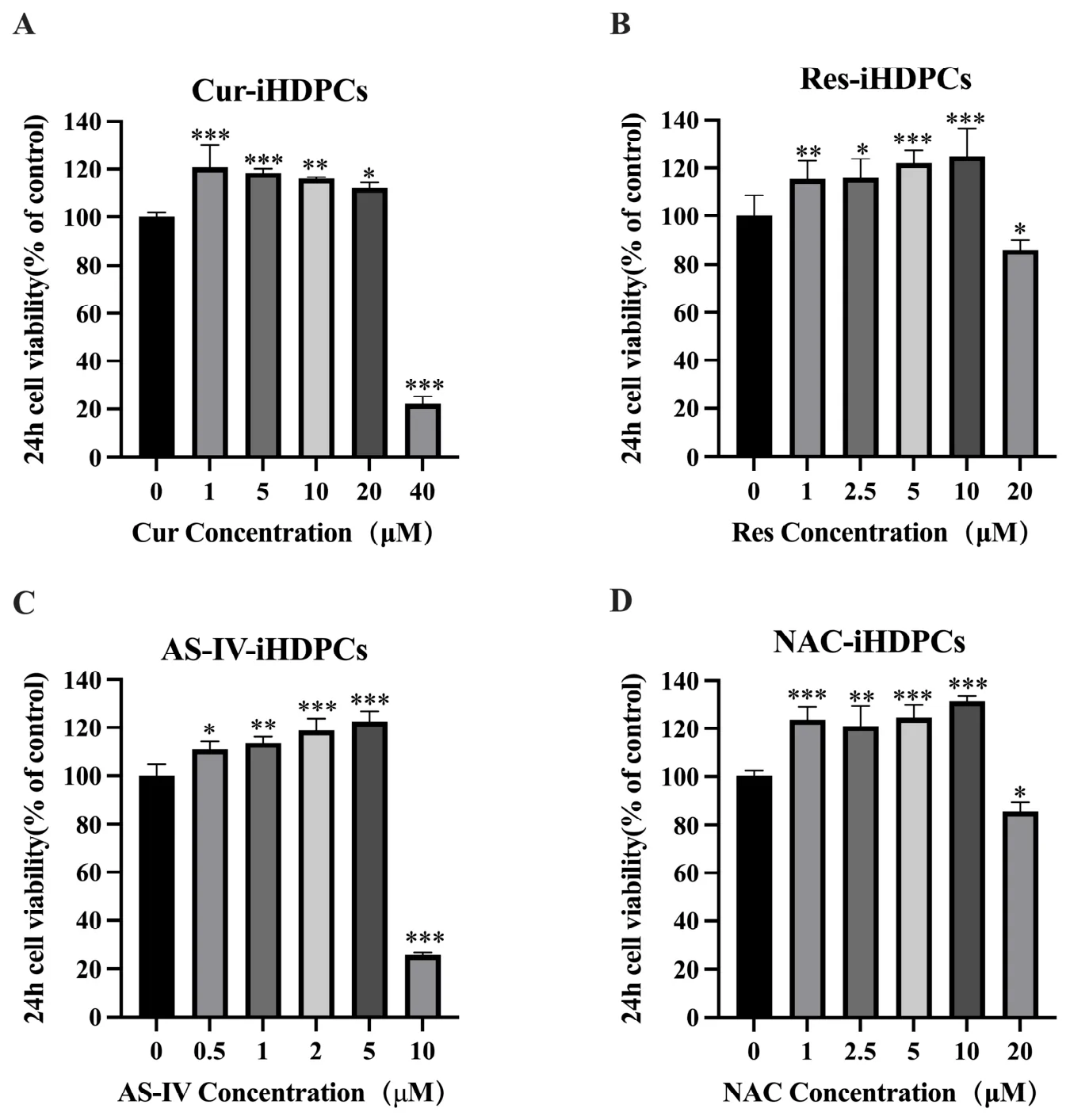
Cytotoxicity of test compounds in iHDPCs. CCK-8 assay showing iHDPC viability after 24 h treatment with graded concentrations of (**A**) Cur (0–40 μM), (**B**) Res (0–20 μM), (**C**) AS-IV (0–10 μM), and (**D**) NAC (0–20 μM). Data are presented as mean ± SD (*n* = 3). * *p* < 0.05, ** *p* < 0.01, *** *p* < 0.001 vs. control.

To establish baseline safety parameters prior to mechanistic evaluation, iHDPCs were treated with graded concentrations of each compound for 24 h. As shown in Figure 5A–D, low to moderate concentrations did not impair cell viability; instead, they slightly promoted proliferation. Based on these toxicity screening data, we defined the non-toxic ranges (viability > 90%) as: Cur 1–20 μM, Res 1–10 μM, and AS-IV 0.5–5 μM.

For subsequent mechanistic studies investigating protective effects against PPD-induced injury, we selected a single representative concentration from within these safe ranges—Cur at 1 μM, Res at 10 μM, and AS-IV at 5 μM, with NAC at 1 mM as the positive control. These concentrations were chosen as standardized conditions to ensure that any observed protective effects on ROS reduction, mitochondrial membrane potential restoration, and apoptosis inhibition are not confounded by intrinsic cytotoxicity, thereby allowing a comparative assessment of their relative pharmacological activities at equitoxic (safe) baseline levels.

At the selected concentrations, AS-IV consistently exhibited the most pronounced protective effects among the three test compounds, as evidenced by its superior ROS-scavenging capacity (approximately 65% reduction vs. injury group) and corresponding molecular docking affinity profile.

### 2.5. All Three Compounds Restore Cell Viability in PPD/H_2_O_2_-Injured iHDPCs

**Figure 6:**
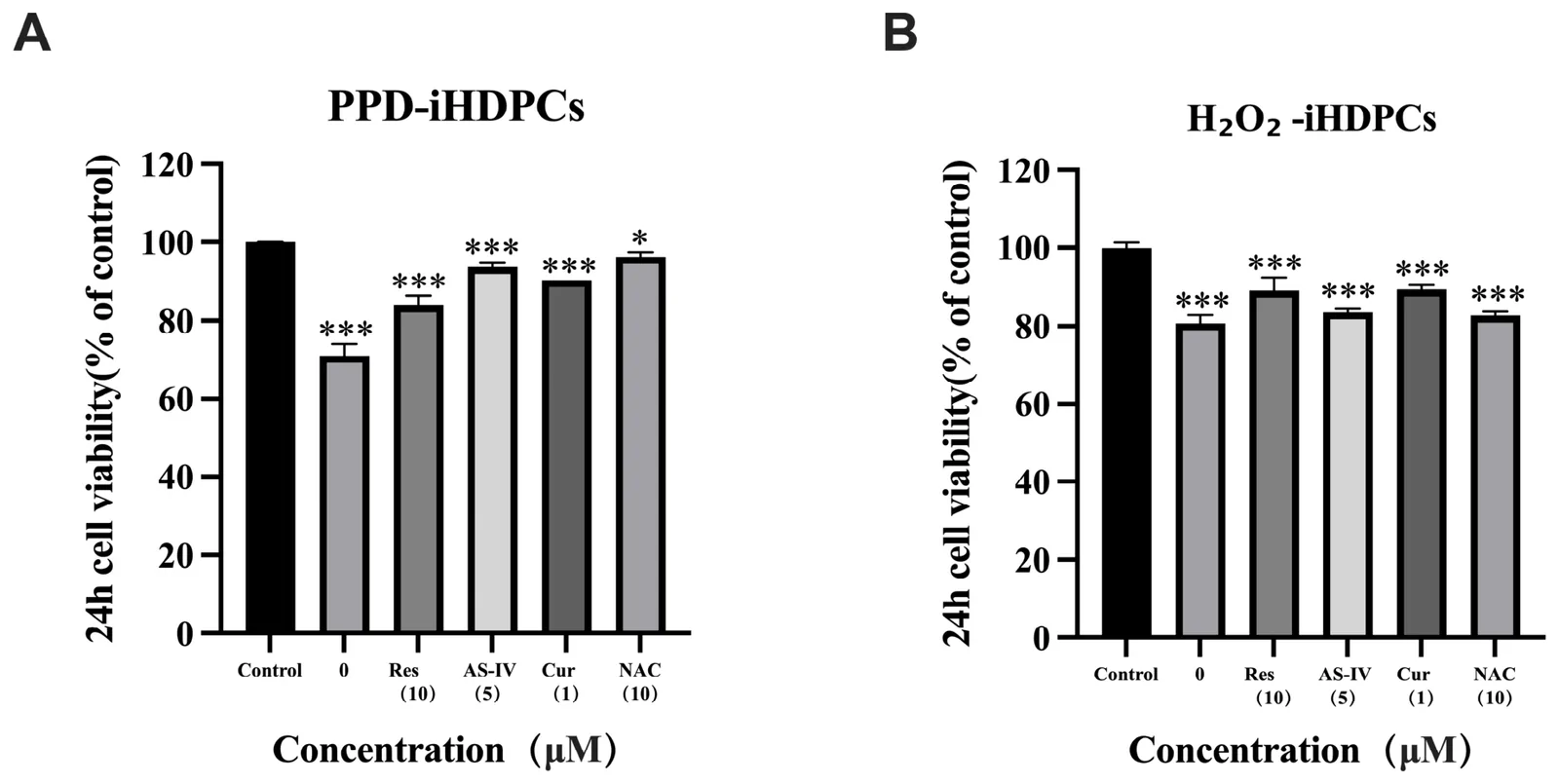
Repair effects of Cur, Res, and AS-IV on PPD/H_2_O_2_-injured iHDPCs. (**A**) Cell viability in the PPD-induced injury model. iHDPCs were exposed to PPD (300 μM) alone or co-treated with Cur (1 μM), Res (10 μM), or AS-IV (5 μM) for 24 h. (**B**) Cell viability in the H_2_O_2_-induced injury model. Cells were exposed to H_2_O_2_ (300 μM) alone or co-treated with Cur (1 μM), Res (10 μM), AS-IV (5 μM), or NAC (1 μM, positive control) for 24 h. Data are presented as mean ± SD. Statistical significance was determined by one-way ANOVA followed by Tukey’s post hoc test. Data are presented as mean ± SD (*n* = 3). * *p* < 0.05, *** *p* < 0.001 vs. the injury-alone group.

The repair effects of the three compounds were evaluated in both PPD-only and PPD + H_2_O_2_ mixed injury models. In the PPD-induced injury model (Figure 6A), PPD treatment (300 μM) significantly reduced cell viability to approximately 80% of the control level (*p* < 0.001). Treatment with Cur (1 μM), Res (10 μM), or AS-IV (5 μM) significantly increased cell viability compared to the PPD-only group. AS-IV showed the strongest repair effect, restoring cell viability to approximately 95% (*p* < 0.05 vs. PPD group). Cur and Res also effectively restored cell viability (both *p* < 0.001 vs. PPD group). In the H_2_O_2_-induced injury model (Figure 6B), H_2_O_2_ treatment (300 μM) significantly reduced cell viability to approximately 75% of the control level (*p* < 0.001). All three compounds significantly restored cell viability, with AS-IV showing the strongest effect among the natural compounds (*p* < 0.001 vs. H_2_O_2_ group), followed by Cur and Res. These results indicate that AS-IV exhibits the strongest repair activity in both injury models, suggesting a superior cytoprotective capacity against oxidative damage in iHDPCs.

### 2.6. All Three Compounds Reduce Intracellular ROS Accumulation

**Figure 7:**
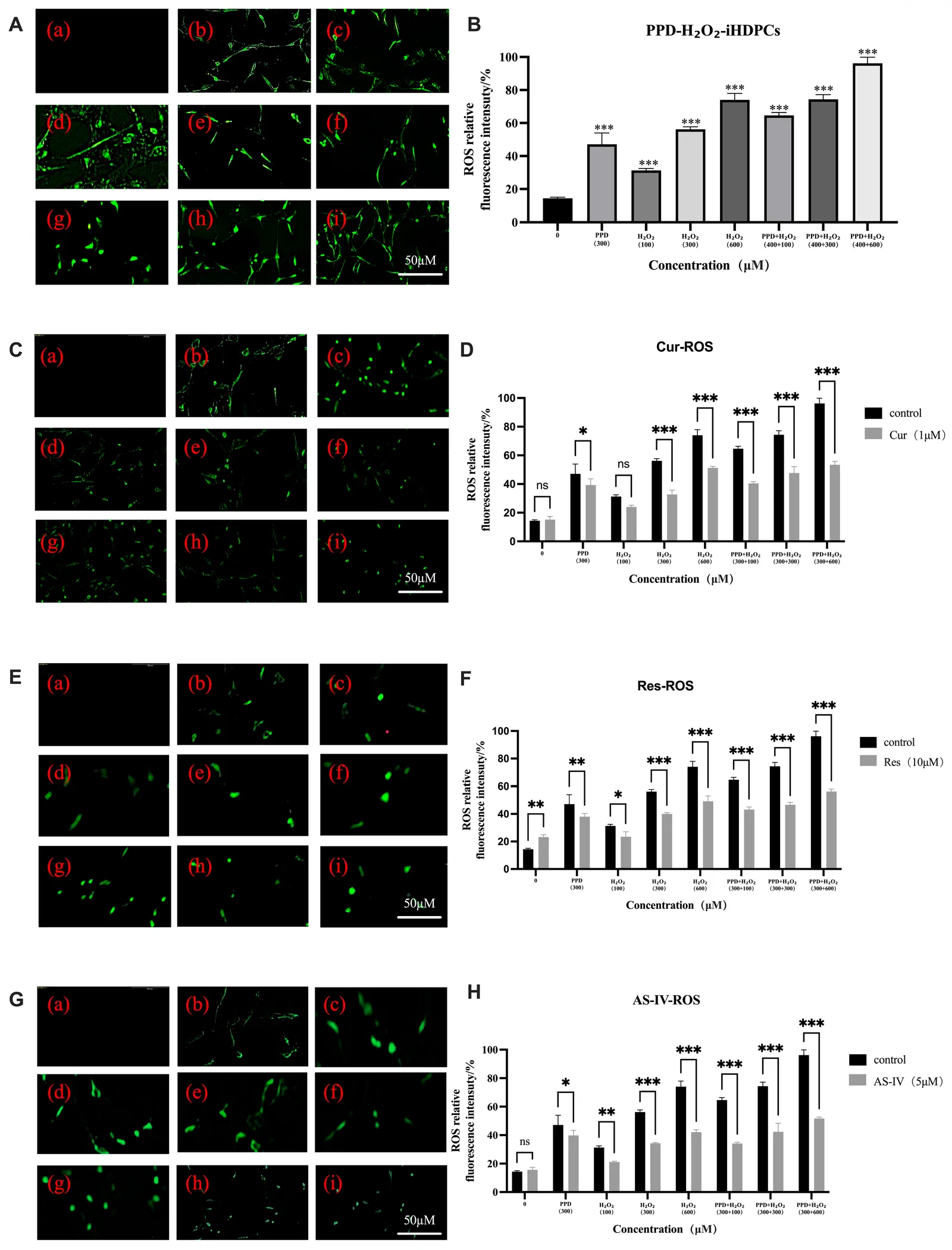
Cur, Res, and AS-IV reduce intracellular ROS levels. (**A**) Fluorescence microscopy images of DCFH-DA-stained iHDPCs showing ROS levels after treatment with PPD (300 μM) and/or H_2_O_2_ (100–600 μM). Scale bar = 50 μM. (**B**) Quantitative analysis of relative fluorescence intensity corresponding to (**A**). (**C**,**E**,**G**) Fluorescence microscopy images showing ROS levels after treatment with Cur (1 μM), Res (10 μM), or AS-IV (5 μM), respectively. (a–i) represent fluorescence microscopy images of intracellular reactive oxygen species (ROS) levels (green fluorescence). (**D**,**F**,**H**) Quantitative analysis of relative fluorescence intensity corresponding to (**C**), (**E**), and (**G**), respectively. All quantitative data are presented as mean ± SD (*n* = 3). * *p* < 0.05, ** *p* < 0.01, *** *p* < 0.001 vs. control.

Intracellular ROS levels were assessed by DCFH-DA fluorescence staining. Treatment with PPD (300 μM) or H_2_O_2_ (100–600 μM) dramatically increased green fluorescence intensity, indicating substantial ROS overproduction. When PPD (300 μM) was combined with H_2_O_2_ (100–600 μM), the fluorescence signal further increased in a concentration-dependent manner, with the PPD + 600 μM H_2_O_2_ group exhibiting the highest signal intensity, suggesting a synergistic oxidative effect between the two agents. Quantitative analysis confirmed that PPD and H_2_O_2_ treatment significantly elevated ROS levels compared to the control group (Figure 7A,B).

Co-treatment with Cur (1 μM) significantly attenuated the fluorescence signal compared to the injury-alone group (Figure 7C). Quantitative analysis confirmed that Cur significantly reduced intracellular ROS levels (* *p* < 0.01 vs. injury group) (Figure 7D). Similarly, treatment with Res (10 μM) markedly decreased the fluorescence intensity (Figure 7E), with quantitative analysis showing a significant reduction in ROS levels (* *p* < 0.01 vs. injury group) (Figure 7F). AS-IV (5 μM) also substantially attenuated the fluorescence signal (Figure 7G), achieving the greatest reduction among the three compounds (* *p* < 0.001 vs. injury group) (Figure 7H). Quantitatively, AS-IV reduced ROS levels by approximately 65% relative to the injury group, followed by Res (approximately 55%) and Cur (approximately 48%). These results suggest that Cur, Res, and AS-IV each possess ROS-scavenging activity in PPD/H_2_O_2_-treated iHDPCs, with AS-IV exhibiting the most prominent effect among the tested compounds. The superior ROS reduction achieved by AS-IV is consistent with its high binding affinity to NRF2 observed in molecular docking, supporting the interpretation that its antioxidant mechanism may involve both direct scavenging and transcriptional activation of endogenous antioxidant defenses. However, this remains to be directly demonstrated, as the present study contains no measurements of NRF2 nuclear translocation, HO-1/NQO1 expression, BAX/Bcl-2 ratio, cytochrome c release, or caspase activation. Therefore, the proposed pathway involvement is inferred rather than established, and future studies should include direct pathway-level validation to confirm target engagement and causal mechanisms.

### 2.7. All Three Compounds Restore Mitochondrial Membrane Potential (ΔΨm)

**Figure 8:**
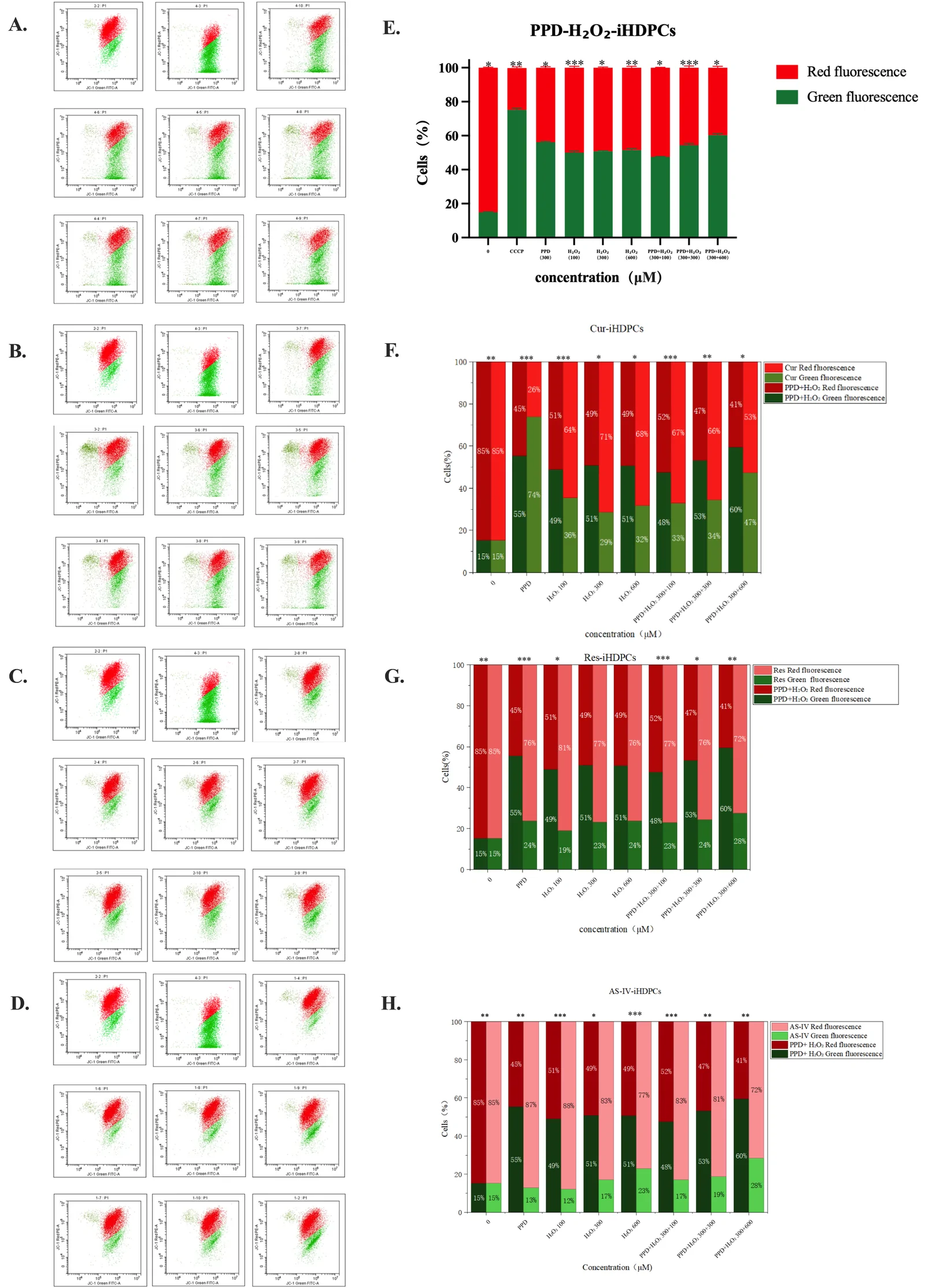
Cur, Res, and AS-IV restore mitochondrial membrane potential (ΔΨm) in PPD/H_2_O_2_-injured iHDPCs. (**A**) Representative flow cytometry plots of JC-1 staining showing the shift from red (JC-1 aggregates, indicating high ΔΨm) to green (JC-1 monomers, indicating low ΔΨm) fluorescence in iHDPCs treated with PPD (300 μM) and/or H_2_O_2_ (100, 300, 600 μM) for 24 h. (**B**–**D**) Representative flow cytometry plots of JC-1 staining in iHDPCs co-treated with PPD/H_2_O_2_ and Cur (1 μM, (**B**)), Res (10 μM, (**C**)), or AS-IV (5 μM, (**D**)). JC-1 staining was used to assess mitochondrial membrane potential (ΔΨm). Red fluorescence corresponds to JC-1 J-aggregates (emission maximum ~590 nm) in polarized mitochondria with high ΔΨm, whereas green fluorescence represents JC-1 monomers (emission maximum ~529 nm) mainly distributed in the cytosol, indicating decreased ΔΨm and depolarization. Reduction of the red/green fluorescence intensity ratio reflects mitochondrial depolarization. (**E**) Quantitative analysis of the red/green fluorescence intensity ratio corresponding to the control and PPD/H_2_O_2_ injury groups in A. (**F**–**H**) Quantitative analysis of the red/green fluorescence intensity ratio corresponding to the Cur (**F**), Res (**G**), and AS-IV (**H**) treatment groups, respectively. A higher red/green ratio indicates intact mitochondrial function. Data are presented as mean ± SD (*n* = 3). Statistical significance was determined by one-way ANOVA followed by Tukey’s post hoc test. * *p* < 0.05, ** *p* < 0.01, *** *p* < 0.001 compared with the PPD/H_2_O_2_ injury group.

Mitochondrial membrane potential (ΔΨm) was measured using JC-1 staining and flow cytometry. In healthy cells, JC-1 forms red fluorescent aggregates (high ΔΨm); upon depolarization, it shifts to green fluorescent monomers (low ΔΨm). Treatment with PPD (300 μM) or H_2_O_2_ (100–600 μM) alone or in combination caused a marked decrease in the red/green fluorescence ratio, reflecting ΔΨm loss. The effect was concentration-dependent, with the PPD + 600 μM H_2_O_2_ group showing the most severe depolarization (Figure 8A). Quantitative analysis confirmed that PPD/H_2_O_2_ injury significantly reduced the red/green ratio compared to the control group (Figure 8E).

Treatment with Cur (1 μM) significantly increased the red/green ratio compared to the PPD + H_2_O_2_ injury group (Figure 8B,F). Res (10 μM) also partially restored ΔΨm (Figure 8C,G). Notably, AS-IV (5 μM) showed the most potent protective effect, restoring the red/green ratio to approximately 75% of the control level (Figure 8D,H). Quantitative comparison across all treatment groups confirmed that AS-IV was the most effective in preserving mitochondrial integrity (*p* < 0.05 vs. Cur and Res groups), followed by Res and Cur. These results demonstrate that Cur, Res, and AS-IV each protect against PPD/H_2_O_2_-induced mitochondrial depolarization in iHDPCs. AS-IV appeared to exhibit a relatively stronger effect among the tested compounds; however, this observation may reflect the specific concentrations used rather than an intrinsic superiority. The preservation of ΔΨm by these compounds, particularly AS-IV, may contribute to their anti-apoptotic effects by preventing mitochondrial outer membrane permeabilization and subsequent release of pro-apoptotic factors, though further validation is required.

### 2.8. All Three Compounds Suppress Apoptosis

**Figure 9:**
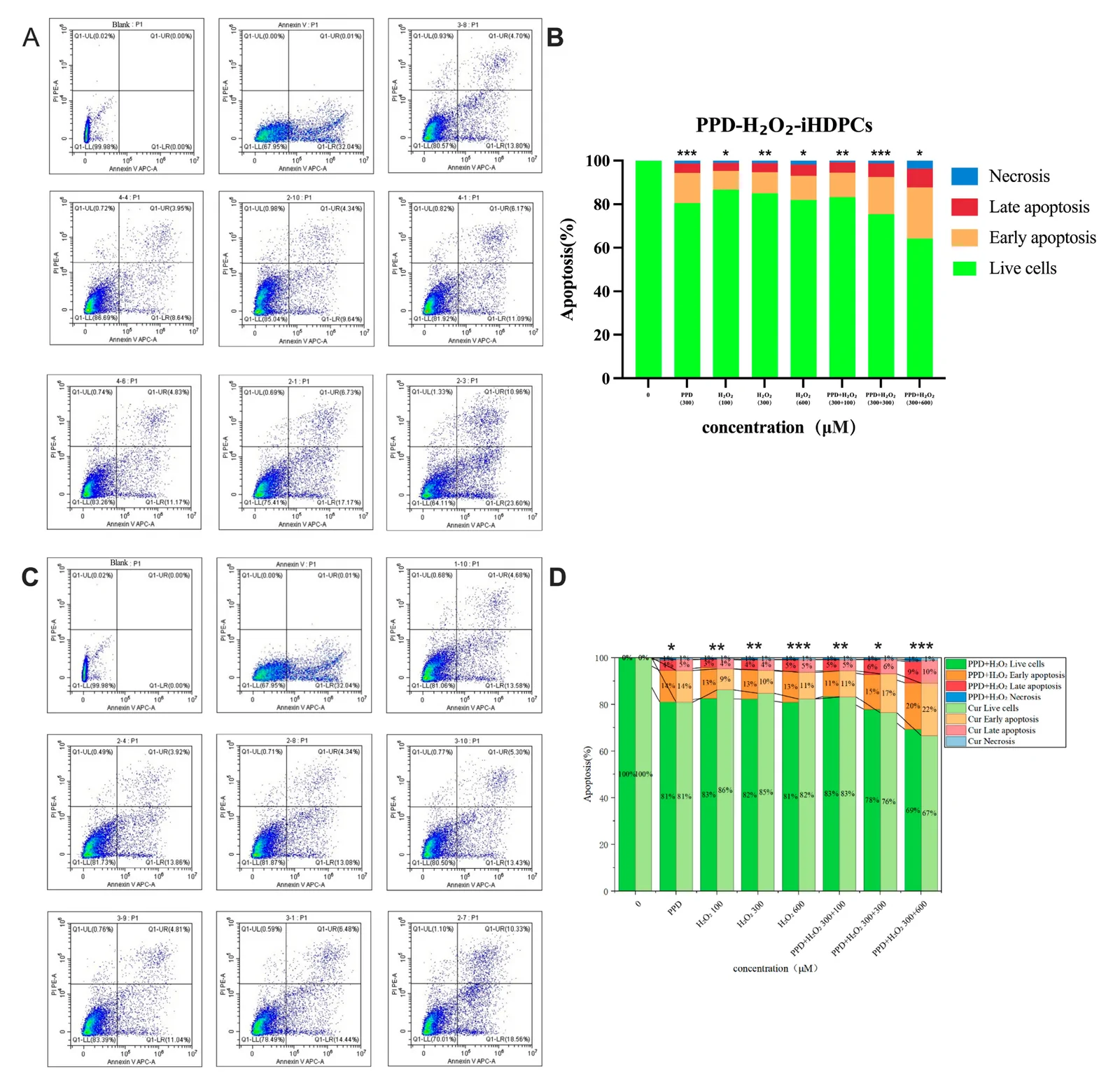

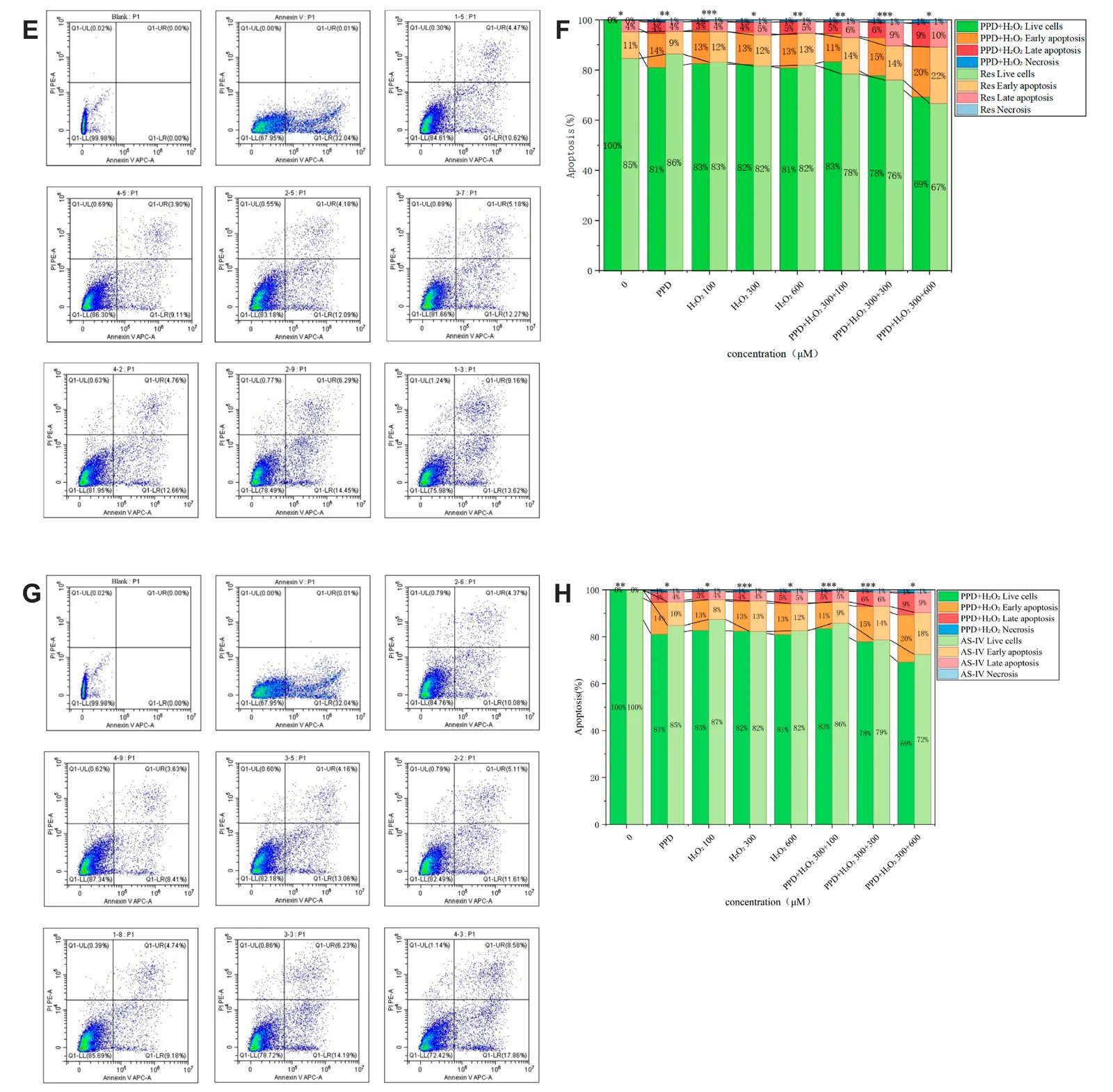
Cur, Res, and AS-IV suppress apoptosis. (**A**) Representative Annexin V-FITC/PI flow cytometry plots of iHDPCs subjected to PPD/H_2_O_2_ injury. (**B**) Quantitative analysis of live, early apoptotic, late apoptotic, and necrotic cell populations in PPD/H_2_O_2_-injured iHDPCs. (**C**) Representative Annexin V-FITC/PI flow cytometry plots of iHDPCs treated with PPD/H_2_O_2_ and Cur (1 μM). (**D**) Quantitative analysis of live, early apoptotic, late apoptotic, and necrotic cell populations in iHDPCs treated with PPD/H_2_O_2_ and Cur (1 μM). (**E**) Representative Annexin V-FITC/PI flow cytometry plots of iHDPCs treated with PPD/H_2_O_2_ and Res (10 μM). (**F**) Quantitative analysis of live, early apoptotic, late apoptotic, and necrotic cell populations in iHDPCs treated with PPD/H_2_O_2_ and Res (10 μM). (**G**) Representative Annexin V-FITC/PI flow cytometry plots of iHDPCs treated with PPD/H_2_O_2_ and AS-IV (5 μM). (**H**) Quantitative analysis of live, early apoptotic, late apoptotic, and necrotic cell populations in iHDPCs treated with PPD/H_2_O_2_ and AS-IV (5 μM). Data are presented as mean ± SD (*n* = 3). Statistical significance was determined by one-way ANOVA followed by Tukey’s post hoc test. * *p* < 0.05, ** *p* < 0.01, *** *p* < 0.001 vs. the injury-alone group.

Apoptosis was quantified by Annexin V-FITC/PI double staining and flow cytometry. Treatment with PPD (300 μM) and H_2_O_2_ (100–600 μM) alone or in combination significantly decreased the percentage of live cells (Annexin V^−^/PI^−^, lower left quadrant) and increased early apoptotic (Annexin V^+^/PI^−^, lower right) and late apoptotic (Annexin V^+^/PI^+^, upper right) populations in a concentration-dependent manner (Figure 9A). Quantitative analysis confirmed that PPD/H_2_O_2_ injury significantly reduced the live cell population and increased the total apoptotic population compared to the control group (Figure 9B).

Treatment with Cur (1 μM) significantly decreased apoptosis and increased the live cell percentage compared to the injury group (Figure 9C,D). Res (10 μM) showed similar protective effects (Figure 9E,F). AS-IV (5 μM) demonstrated the strongest anti-apoptotic activity, reducing the total apoptotic population (early + late) from approximately 45% in the injury group to approximately 18% (Figure 9G,H). Quantitative analysis across all groups confirmed that all three compounds significantly suppressed PPD/H_2_O_2_-induced apoptosis (* *p* < 0.05 for Cur and Res; * *p* < 0.05, ** *p* < 0.01, *** *p* < 0.001 for AS-IV), with AS-IV showing the best performance. These results demonstrate that Cur, Res, and AS-IV protect iHDPCs from PPD/H_2_O_2_-induced apoptosis via the OS–mitochondrial pathway, with AS-IV exhibiting superior anti-apoptotic efficacy consistent with its strongest ROS scavenging and mitochondrial protective effects.

## 3. Discussion

The selection of Cur, Res, and AS-IV in this study was based on a combination of their natural origin, structural diversity, and converging mechanistic relevance to oxidative stress–mitochondrial apoptosis. From a cosmetic and consumer perspective, there is a growing demand for naturally derived alternatives to synthetic additives in hair care products, driven by safety concerns and regulatory trends favoring plant-based ingredients with established toxicological profiles. All three compounds are naturally occurring small molecules—curcumin is a hydrophobic polyphenol derived from Curcuma longa (turmeric); resveratrol is a stilbene polyphenol produced by various plants, including grapes and Polygonum cuspidatum, in response to environmental stress; and AS-IV is a triterpene saponin isolated from the root of Astragalus membranaceus, a herb widely used in traditional medicine. Beyond their natural sourcing, these agents were chosen because they represent distinct chemical classes that independently target the oxidative stress–mitochondrial apoptosis axis through complementary mechanisms: curcumin activates the NRF2/ARE pathway and inhibits NF-κB–mediated inflammatory signaling [19,20,21,22]; resveratrol activates SIRT1, thereby deacetylating and stabilizing p53 and FOXO3 to promote mitochondrial homeostasis and resistance to oxidative damage [23]; and AS-IV has been reported to inhibit mitochondrial permeability transition pore opening, stabilize mitochondrial membrane potential, and modulate the PI3K/AKT and MAPK cascades to suppress intrinsic apoptosis [24]. This convergence on overlapping yet non-redundant nodes within the same pathway provided a strong rationale for directly comparing their protective effects against PPD-induced follicular injury within a single, controlled experimental framework.

Against this background, the present study evaluated the reparative effects of these three compounds against PPD-induced oxidative damage in iHDPCs, with phenotypic readouts linked to the oxidative stress–mitochondrial apoptosis axis. By integrating network pharmacology, molecular docking, and in vitro functional assays, we found that all three compounds attenuated PPD-induced injury in a manner consistent with modulation of key nodes within this pathway. Among the three, AS-IV showed numerically higher efficacy than Cur and Res across the majority of tested functional parameters; however, these differences were not uniformly statistically significant across all assays, and the apparent superiority of AS-IV should be interpreted as a trend rather than a definitive conclusion. The relative performance of these compounds may depend on experimental variables including cell model, exposure duration, and concentration range, and firm conclusions regarding comparative efficacy will require validation through protein-level assays, dose–response analyses across broader concentration windows, and physiologically relevant models. Based on network pharmacology, we predicted that these three compounds have 166 intersection targets with hair follicle damage. The PPI network screening identified 35 targets that are located at central positions within the OS and mitochondrial apoptosis pathways. These targets are enriched in biological processes such as “negative regulation of apoptosis” and “positive regulation of cell proliferation,” which are relevant to the pathology of PPD-induced hair follicle damage. KEGG pathway enrichment includes PI3K-Akt and MAPK signaling, both of which have been previously associated with PPD-induced cellular stress and survival regulation in dermal papilla cells. Notably, these three compounds converge on the NRF2–BAX–Bcl-2 regulatory axis, a known decision point in OS-mediated mitochondrial apoptosis. However, these in silico predictions are purely computational and await direct experimental verification at the protein level.

Molecular docking provided a structural rationale for the differential activities observed. The binding affinity heatmap revealed that Cur, Res, and AS-IV all exhibited their strongest binding to P-AKT, with AS-IV showing the highest overall affinity (−9.4 kcal/mol), followed by Cur (−8.0 kcal/mol) and Res (−7.8 kcal/mol). AS-IV also demonstrated strong binding to NRF2 (−8.3 kcal/mol), BAX (−7.7 kcal/mol), and HO-1 (−7.4 kcal/mol), while Cur and Res showed moderate preferences for NRF2 (−7.1 and −6.4 kcal/mol, respectively). These results are consistent with previous studies reporting AS-IV activation of the NRF2–HO-1 axis in retinal ganglion cells [11], and extend this observation by quantitatively demonstrating its high target engagement at the molecular level. The extensive hydrogen bond network formed by AS-IV with P-AKT residues ASP-293, THR-292, and GLY-312 likely contributes to its high binding stability. Cur formed hydrogen bonds with key residues across multiple targets, including GLU-193, GLY-312, and ASP-293 in P-AKT, ASN-507 and ARG-504 in NRF2, and GLN-52 and LYS-64 in BAX, suggesting its mechanism may involve multi-target modulation. Res displayed stable binding across all five targets, with a combination of hydrophobic interactions and hydrogen bonds at P-AKT (e.g., ALA-232, LYS-181, ASP-293) and hydrogen bonds with ASN-507 and GLN-26 in NRF2.

The experimental verification confirmed the functional correlations of these predictions. Both PPD and H_2_O_2_ reduced the survival rate of iHDPCs in a concentration-dependent manner, and the IC_20_ values of both substances were approximately 300 μM. It is notable that this concentration is far below the 2% (*w*/*w*) regulatory limit of PPD in cosmetics, yet it still triggered significant excessive production of reactive oxygen species (ROS) and cell apoptosis [25]. The underlying reason for this is that our experiments were conducted at the cellular level, while in reality, the exposure scenario involves human scalp tissue rather than direct application to isolated cells.

When evaluating the protective effects, all three compounds significantly restored cell survival in both damage models. In the PPD-induced model, AS-IV showed the most pronounced repair effect among the tested compounds at the selected concentrations, increasing the survival rate from approximately 80% of the damaged control group to approximately 95%. This observation is consistent with its highest overall binding affinity to P-AKT and strong NRF2 engagement observed in docking, as well as its most efficient ROS-scavenging capacity. In the H_2_O_2_-induced model, the positive control NAC demonstrated the strongest protective effect, consistent with its mechanism as a direct ROS scavenger and glutathione precursor. It is noteworthy that at 5 μM, the ROS clearance rate achieved by AS-IV (65%) was higher than that of 1 mM NAC (52%). This difference cannot be explained solely by direct free radical scavenging and may involve additional signaling pathways, such as activation of NRF2 to upregulate endogenous antioxidant enzymes including HO-1, NQO1, and glutathione S-transferase [26]. This “indirect antioxidant” mode has been described for various phytochemicals and is considered more sustainable than direct clearance, as it may restore the cell’s intrinsic redox homeostasis. However, direct measurements of NRF2 nuclear translocation, HO-1/NQO1 expression, or caspase activation are still required to confirm this proposed mechanism.

The JC-1 assay results further corroborated the mitochondrial protective effects at the functional level. PPD and H_2_O_2_ exposure caused marked depolarization of mitochondrial membrane potential, consistent with the established role of mitochondria as both a primary source and primary target of OS. AS-IV restored ΔΨm to approximately 75% of the control level, outperforming Cur and Res. This effect is phenotypically consistent with a possible shift in the BAX/Bcl-2 balance—by potentially stabilizing Bcl-2 function while inhibiting BAX oligomerization, AS-IV may tip the balance toward mitochondrial integrity, which would prevent cytochrome c release and subsequent caspase activation. However, because we did not directly measure BAX/Bcl-2 protein ratio, cytochrome c release, or caspase activation, this mechanistic interpretation remains correlative and requires biochemical validation. The importance of the BAX/Bcl-2 balance in determining dermal papilla cell fate has been demonstrated in studies on androgenetic alopecia and chemotherapy-induced hair loss [27], and our findings are consistent with extending this concept to chemically induced follicular damage, but do not prove it.

Annexin V/PI apoptosis detection provides functional evidence of anti-apoptotic effects at the cellular level [28]. After PPD and H_2_O_2_ treatments, the proportion of apoptotic cells increased in a concentration-dependent manner, confirming that OS-associated mitochondrial dysfunction is a dominant pathway leading to cell death in this model. AS-IV reduced the total proportion of apoptotic cells in the injured group from approximately 45% to approximately 18%, indicating its strongest inhibitory effect among the three compounds in terms of apoptosis phenotype [29]. The downward trend of apoptosis correlates with the ROS and ΔΨm data, forming a coherent picture that is consistent with the OS–mitochondrial apoptosis cascade, but this correlation does not establish causality without direct molecular readouts.

Importantly, the central focus of the present investigation was restricted to the characterization of cellular phenotypic responses to Cur, Res, and AS-IV under PPD-induced oxidative stress, and to the exploration of whether these responses are correlated with the OS–mitochondrial apoptosis pathway. Our experimental design was intentionally constructed as a reductionist in vitro model to isolate and systematically dissect the functional readouts along this axis, rather than to simulate the full complexity of topical product application. Consequently, all concentration selections, exposure protocols, and outcome measurements were optimized for phenotypic clarity and comparative pharmacological profiling, not for formulation compatibility or biopharmaceutical performance. We recognize that the translation of these phenotypic insights into practical hair dye applications will require a separate and equally rigorous body of research addressing formulation design, skin permeation, and product stability. Within the scope of this study, however, our primary contribution is the identification of a consistent set of protective phenotypes and the prioritization of AS-IV as the most promising candidate for subsequent formulation-oriented development, based on its superior functional performance.

This study has several limitations. First, the network pharmacology and docking results are computational and require protein-level validation (e.g., Western blot for NRF2, HO-1, NQO1, BAX/Bcl-2, cytochrome c, caspase-3) to confirm target engagement. Second, iHDPCs may not fully recapitulate primary cell physiology; 3D organoids or organ-on-chip models would improve physiological relevance. Third, the acute 24 h exposure does not reflect chronic, repeated hair-dye exposure in real-world settings. Fourth, NAC was chosen as a mechanistic positive control; future comparisons with cosmetic antioxidants (vitamin E, ascorbic acid) would strengthen translational relevance. Fifth, topical bioavailability of Cur, Res, and AS-IV remains unknown due to skin-barrier limitations; the tested concentrations (1–10 μM) established in vitro dose–response relationships but were not intended to predict in vivo tissue levels. Translation will require advanced delivery systems and ex vivo permeation studies. Finally, long-term safety and sensitization potential require further evaluation.

## 4. Materials and Methods

### 4.1. Chemicals and Reagents

PPD (purity ≥ 99%, CAS 106-50-3, molecular weight: 108.14 g/mol) and hydrogen peroxide (H_2_O_2_, 3% (*w*/*w*) solution in H_2_O) were purchased from Yuanye Biotechnology Co., Ltd. (Shanghai, China). Cur (purity ≥ 98%, CAS 458-37-7), Res (purity ≥ 99%, CAS 501-36-0), AS-IV (purity ≥ 98%, CAS 84687-43-4), and N-acetylcysteine (NAC, purity ≥ 99%, CAS 616-91-1) were also obtained from Yuanye Biotechnology Co., Ltd. (Shanghai, China). Stock solutions were prepared as follows: PPD was dissolved in DMSO at a concentration of 1 M; Cur, Res, and AS-IV were dissolved in DMSO at concentrations of 10 mM, 50 mM, and 10 mM, respectively; NAC was dissolved in sterile water at a concentration of 10 mM. All stock solutions were stored at −20 °C and diluted freshly in culture medium before use. For PPD treatments at a final concentration of 300 μM, 3.0 μL of the 1 M stock and 7.0 μL of pure DMSO were added to approximately 9.99 mL of complete medium (total volume 10 mL), yielding a final DMSO concentration of 0.1% (*v*/*v*). A vehicle control containing the same final DMSO concentration (0.1% *v*/*v*) without PPD was included in all experiments. The final DMSO concentration in the culture medium did not exceed 0.1% (*v*/*v*), which was previously confirmed to have no effect on cell viability.

### 4.2. Cell Culture

The iHDPCs were obtained from Fuheng Biotechnology Co., Ltd. (Shanghai, China). The cells were cultured in complete medium specifically formulated for iHDPCs at 37 °C in a humidified atmosphere containing 5% CO_2_. The culture medium was replaced every 2–3 days, and the cells were passaged using 0.25% trypsin-EDTA (Gibco, catalog number 25200056, Waltham, MA, USA) upon reaching 80–90% confluence. iHDPCs from passages 5 to 15 were used for all experiments.

### 4.3. Network Pharmacology Analysis

Compound targets for Cur, Res, and AS-IV were retrieved from SwissTargetPrediction (http://www.swisstargetprediction.ch/), PharmMapper (http://www.lilab-ecust.cn/pharmmapper/) (accessed on 1 February 2026), and BATMAN-TCM (http://bionet.ncpsb.org.cn/batman-tcm/) (accessed on 1 February 2026). Only targets with probability > 0.7 were retained. Hair follicle injury-associated targets were collected from GeneCards (https://www.genecards.org/) and DisGeNET (https://www.disgenet.org/) using the keywords “hair follicle injury,” “dermal papilla injury,” “PPD toxicity,” and “OS hair.” The intersection of compound targets and disease targets was identified using Venny 2.1 (https://bioinfogp.cnb.csic.es/tools/venny/) (accessed on 1 February 2026). Protein–protein interaction (PPI) networks were constructed using the STRING database (https://string-db.org/, version 11.5) with a confidence score threshold of 0.4 and visualized using Cytoscape (version 3.9.1). Core targets were identified using the CytoHubba plugin with degree, betweenness, and closeness centrality algorithms; the top 35 targets were considered core targets. Gene Ontology (GO) functional enrichment and Kyoto Encyclopedia of Genes and Genomes (KEGG) pathway enrichment analyses were performed using DAVID (https://david.ncifcrf.gov/, version 6.8). A *p*-value < 0.05 and a count ≥ 2 were considered statistically significant.

### 4.4. PPI Network Construction and Core Target Screening

The 166 intersection targets were imported into the STRING database (version 11.5) to construct a protein–protein interaction (PPI) network with a confidence score threshold of 0.4. To identify hub targets, topological filtering was performed based on three centrality parameters: Degree > 30.229, Betweenness > 156.108, and Closeness > 0.0032. This filtering yielded 35 core targets, including well-established regulators of OS and apoptosis such as NRF2, BAX, Bcl-2, HO-1, and P-AKT. PPI network visualization and topology analysis of the 35 core targets were performed using Cytoscape software (version 3.9.1).

### 4.5. Molecular Docking

Three-dimensional structures of core target proteins were retrieved from the RCSB Protein Data Bank (https://www.rcsb.org/) using the following PDB IDs: NRF2 (AF-Q16236), BAX (AF-Q07812), Bcl-2 (4LVT), HO-1 (1N45), and P-AKT (1O6K). Protein structures were prepared using AutoDockTools (version 1.5.6) by removing water molecules, adding hydrogen atoms, and optimizing hydrogen bonds. Three-dimensional structures of Cur, Res, AS-IV, and NAC were downloaded from PubChem (https://pubchem.ncbi.nlm.nih.gov/) and converted to PDB format using Open Babel (version 2.4.1). Molecular docking was performed using AutoDock Vina (version 1.1.2). The grid box was centered on the predicted binding site with dimensions of 25 Å × 25 Å × 25 Å for each protein, with an exhaustiveness parameter of 8. The best docking pose was selected based on the lowest binding energy (kcal/mol). Visualization of docked complexes was performed using PyMOL (version 2.5, Schrödinger, LLC, New York, NY, USA). A binding energy ≤ −7.0 kcal/mol was considered indicative of strong binding affinity.

### 4.6. Cell Viability Assay (CCK-8)

Cell viability was evaluated using the Cell Counting Kit-8 (CCK-8, Beyotime Biotechnology, catalog number C0037, Shanghai, China). iHDPCs were seeded into 96-well plates (Corning, catalog number 3599, Corning, NY, USA) at a density of 1 × 10^4^ cells per well and allowed to attach overnight. Following a 24 h incubation period, the cells were subjected to various treatments to assess cytotoxicity and compound efficacy. To determine the half-maximal inhibitory concentration (IC_50_) and the 20% inhibitory concentration (IC_20_), cells were exposed to graded concentrations of PPD (0–800 μM) or H_2_O_2_ (0–800 μM) for 24 h. To evaluate the effects of candidate compounds on basal cell metabolic activity, cells were treated with increasing concentrations of Cur (0–50 μM), Res (0–50 μM), AS-IV (0–20 μM), or NAC (0–20 μM) for 24 h, and the concentration ranges without marked suppression of metabolic activity were identified. In the intervention experiment, the cells were subjected to 1-h oxidative damage under three conditions: PPD (300 μM, IC_20_), H_22_ (100, 300 or 600 μM), and PPD (300 μM) combined with different concentrations of H_2_O_2_ (100–600 μM). After 1-h damage, the culture medium was aspirated, the cells were washed with PBS, and then replaced with fresh medium containing Cur (1μM), Res (10 μM), AS-IV (5 μM) or NAC (1 μM) for 24-h recovery. This experimental design, which maintained a constant PPD level while varying the H_2_O_2_ concentration, was intended to mimic the graded OS environment that dermal papilla cells may encounter during repeated or prolonged hair dyeing procedures. At the end of the treatment period, 10 μL of CCK-8 solution was added to each well, and the plates were incubated for an additional 2 h at 37 °C in a humidified atmosphere. Absorbance was recorded at 450 nm with a reference wavelength of 650 nm using a microplate reader (Multiskan GO, Thermo Fisher Scientific, Waltham, MA, USA). Cell viability was calculated using the following formula:

$$\mathrm{Cell}\mathrm{viability}\left(\%\right)=\frac{\left(OD_{\mathrm{treatment}}-OD_{\mathrm{blank}}\right)}{\left(OD_{\mathrm{control}}-OD_{\mathrm{blank}}\right)}\times 100\%$$

The IC_20_ and IC_50_ values were derived by fitting dose–response curves using GraphPad Prism 9.0 (GraphPad Software, San Diego, CA, USA). All experiments were conducted in triplicate and repeated on at least three independent occasions.

### 4.7. Intracellular ROS Measurement (DCFH-DA)

Intracellular reactive oxygen species (ROS) levels were detected using the DCFH-DA fluorescent probe (Beyotime Biotechnology, catalog number S0033S, Shanghai, China). iHDPCs were seeded into 12-well plates (Corning, catalog number 3524, Corning, NY, USA) at a density of 2 × 10^5^ cells per well and treated as described above for 24 h. After treatment, cells were washed twice with PBS and incubated with 10 μM DCFH-DA in serum-free medium for 30 min at 37 °C in the dark. Cells were then washed three times with PBS. Fluorescence images were captured using a fluorescence microscope (Olympus IX73, Tokyo, Japan) at 20× magnification (excitation 488 nm, emission 525 nm). Fluorescence intensity was quantified using ImageJ software (version 1.54r, National Institutes of Health, Bethesda, MD, USA).

### 4.8. Mitochondrial Membrane Potential (ΔΨm) Detection (JC-1)

Mitochondrial membrane potential (ΔΨm) was measured using the JC-1 assay kit (Beyotime Biotechnology, catalog number C2006, Shanghai, China) [30]. iHDPCs were seeded into 12-well plates (Corning, catalog number 3516, Corning, NY, USA) at a density of 2.0 × 10^5^ cells per well and treated for 24 h as described above. Cells were harvested using trypsin without EDTA, washed twice with phosphate-buffered saline (PBS), and incubated with 500 μL of JC-1 working solution for 20 min at 37 °C in the dark. After washing twice with JC-1 staining buffer, cells were resuspended in 300 μL of PBS and analyzed using a flow cytometer (Accuri C6, BD Biosciences, Franklin Lakes, NJ, USA). JC-1 aggregates (red fluorescence, indicating healthy mitochondria with high ΔΨm) were detected in the FL2 channel (excitation 488 nm, emission 585 nm), while JC-1 monomers (green fluorescence, indicating depolarized mitochondria) were detected in the FL1 channel (excitation 488 nm, emission 530 nm). For each sample, 20,000 events were collected, and the red/green fluorescence ratio was calculated as an indicator of ΔΨm.

### 4.9. Apoptosis Detection (Annexin V-FITC/PI)

Apoptosis was quantified using the Annexin V-FITC/PI Apoptosis Detection Kit (Beyotime Biotechnology, catalog number C1062S, Shanghai, China). iHDPCs were seeded into 6-well plates (Corning, catalog number 3516, Corning, NY, USA) at a density of 5 × 10^5^ cells per well and treated for 24 h as described above. Cells were harvested using trypsin without EDTA, washed twice with ice-cold phosphate-buffered saline (PBS), and resuspended in 500 μL of 1× binding buffer at a concentration of 1 × 10^6^ cells/mL. Then, 5 μL of Annexin V-FITC and 5 μL of propidium iodide (PI) were added to 100 μL of the cell suspension (1 × 10^5^ cells) and incubated for 15 min at room temperature in the dark. Immediately before flow cytometric analysis, 400 μL of 1× binding buffer was added to each sample. Samples were analyzed within 1 h using a flow cytometer (Accuri C6, BD Biosciences, Franklin Lakes, NJ, USA). Cell populations were classified as follows: live cells (Annexin V^−^/PI^−^, lower left quadrant), early apoptotic cells (Annexin V^+^/PI^−^, lower right quadrant), late apoptotic cells (Annexin V^+^/PI^+^, upper right quadrant), and necrotic cells (Annexin V^−^/PI^+^, upper left quadrant). The total apoptosis rate was calculated as (early apoptotic cells + late apoptotic cells)/total cells × 100%.

### 4.10. Statistical Analysis

All experiments were performed at least three times (*n* = 3 biological replicates). Data are presented as mean ± standard deviation (SD). Statistical analyses were performed using GraphPad Prism (version 9.0, GraphPad Software, San Diego, CA, USA). Comparisons among multiple groups were performed using one-way analysis of variance (ANOVA) followed by Tukey’s post hoc test for multiple comparisons. A *p*-value < 0.05 was considered statistically significant. Significance levels are indicated as: * *p* < 0.05, ** *p* < 0.01, *** *p* < 0.001.

## 5. Conclusions

This study demonstrates that Cur, Res and AS-IV can effectively protect iHDPCs from oxidative damage induced by PPD and subsequent apoptosis. Although our research results (such as the upregulation of Bcl-2 expression, the downregulation of Bax and cleaved caspase-3 expression) suggest that the OS–mitochondrial apoptosis pathway may be involved in this protective mechanism, these results are helpful for us to better understand the hair follicle damage caused by PPD and provide preclinical evidence for the development of “combined dyeing and care”-type hair dyeing preparations, which can combine efficient coloring with protective functions for active hair follicles. By reducing the subclinical oxidative damage accumulated during repeated hair dyeing, such preparations are expected to reduce the long-term risk of hair follicle dysfunction, thereby addressing the common overlooked safety issues in the current regulatory framework for hair dyeing. Future research should focus on in vivo validation, comprehensive mechanism studies, preparation development, and long-term safety assessment to convert these promising in vitro research results into practical benefits for consumers.

## Data Availability

The raw data supporting the conclusions of this article will be made available by the authors on reasonable request.

## Abbreviations

The following abbreviations are used in this manuscript:

ANOVA	analysis of variance
AS-IV	Astragaloside-IV
BAX	Bcl-2-associated X protein
Bcl-2	B-cell lymphoma 2
CCK-8	Cell Counting Kit-8
Cur	Curcumin
DCFH-DA	2′,7′-dichlorodihydrofluorescein diacetate
DMSO	dimethyl sulfoxide
GO	Gene Ontology
H_2_O_2_	hydrogen peroxide
HO-1	heme oxygenase-1
IC_20_	20% inhibitory concentration
IC_50_	half-maximal inhibitory concentration
iHDPCs	immortalized human dermal papilla cells
JC-1	5,5′,6,6′-tetrachloro-1,1′,3,3′-tetraethylbenzimidazolocarbocyanine Iodide
KEGG	Kyoto Encyclopedia of Genes and Genomes
MAPK	mitogen-activated protein kinase
NAC	N-acetylcysteine
NRF2	nuclear factor erythroid 2-related factor 2
OS	oxidative stress
P-AKT	phosphorylated protein kinase B
PBS	phosphate-buffered saline
PI	propidium iodide
PI3K	phosphoinositide 3-kinase
PPD	p-phenylenediamine
PPI	protein–protein interaction
Res	resveratrol
ROS	reactive oxygen species
SD	standard deviation
ΔΨm	mitochondrial membrane potential
